# Clinical outcomes and quality of life after total hip arthroplasty in adult patients with a history of infection of the hip in childhood: a mid-term follow-up study

**DOI:** 10.1186/s13018-019-1074-4

**Published:** 2019-02-01

**Authors:** Yue Luo, Zhouyuan Yang, Releken Yeersheng, Donghai Li, Pengde Kang

**Affiliations:** Department of Orthopedics, West China Hospital, Sichuan University, 37# Wainan Guoxue Road, Chengdu, 610041 Sichuan People’s Republic of China

**Keywords:** Previous infection, Total hip arthroplasty, Complication, Infection recurrence, Efficacy

## Abstract

**Background:**

Total hip arthroplasty for adult patients with a history of infection of the hip in childhood could be a more technically demanding procedure due to complicated anatomy and the possibility of reinfection. Here, we conducted a mid-term analysis of clinical outcomes in such patients after primary cementless total hip arthroplasty (THA).

**Methods:**

We reviewed 101 patients (101 hips; 51 men; mean age, 52.3 years) who underwent cementless THA between 2008 and 2015, at a mean of 24 years (range, 11–43) since the resolution of childhood hip infection. Patients were followed up for a mean of 6.1 years (range, 2.1–9.6). Clinical outcomes and quality of life after THA were assessed at final follow-up.

**Results:**

No cases of infection were reported during the follow-up, and patients showed significant improvement in Harris Hip Score, for which the mean score increased from 48.5 to 90 points; the modified Merle d’Aubigne and Postel (MAP) Hip Score; the Hip Dysfunction and Osteoarthritis Outcome Score; the SF-12; and mean limb length discrepancy, which decreased from 3.4 to 1.1 cm. During follow-up, four cases of prosthesis dislocation, three of transient sciatic paralysis, seven of femoral fracture, five of heterotopic ossification, and 19 of osteolysis were recorded. Revision surgery was performed for two patients, one for isolated loosening of the acetabular component and another for loosening of the femoral stem.

**Conclusion:**

Cementless THA can effectively treat patients with a quiescent period of infection of the hip of more than 10 years, resulting in good functional outcomes and fewer complications. Risk of infection recurrence after THA in these patients seems extremely low.

## Background

Septic arthritis of the hip is the most common septic arthritis in children, with an incidence of about 1:20,000 in developing countries [1]. The prognosis depends on prompt diagnosis and appropriate treatment, such as surgical decompression and proper antibiotic use. If not treated promptly, septic arthritis of the hip may cause anatomic deformation of various bone and soft tissue structures, which can cause pain from secondary arthritis. Performing total hip arthroplasty (THA) in pediatric patients with septic arthritis of the hip is challenging because of their young age, the anatomical abnormalities of bone and soft tissue, and potential risk of postoperative deep infection. Various special surgical techniques have been designed to achieve THA in severely dysplastic hips in this subset of patients, but they are associated with relatively high rates of complications, including intraoperative femoral fractures, recurrent infection, mechanical loosening, and revisions [2–9].

Several studies suggest that THA can provide good prognosis if performed sufficiently long after resolution of hip infection, with rates of recurrence of 0–9.5% [4, 6, 10, 11]. The present study analyzed recurrence and other outcomes in patients who underwent primary cementless THA more than 10 years after resolution of hip infection.

## Methods

### Patient selection

This retrospective study was approved by the relevant Institutional Review Board. From February 2008 to April 2015, a total of 105 cases (108 hips) with osteoarthritis secondary to hip septic infection were treated with cementless total hip replacement in our hospital. The inclusion criteria are as follows: hip osteoarthritis and a clear history of acute pyogenic infection of the hip joint, such as acute hip pain, high fever, sinus formation or the corresponding sinus scar formation, or surgical incision and drainage scar. Exclusion criteria are as follows: osteoarthritis due to hip tuberculosis or fungal infection. The diagnosis of osteoarthritis in 101 cases was referred to the American College of Rheumatology diagnostic criteria for osteoarthritis [12]. The types of pathogens are *Staphylococcus aureus* (80 hips), *Streptococcus* (9 hips), and unknown types of pathogens (12 hips). The mean age of the patient at the time of infection was 9.6 years (range, 7–12). All these patients were considered for enrollment in our study. The indication for total hip replacement is severe pain that does not respond to conservative treatment, resulting in stiffness, limping, and poor quality of life.

### Patient assessment

Preoperative and postoperative clinical evaluations, surgical data, and imaging findings were examined. Clinical evaluations were conducted at 2 weeks, 3 months, and 6 months after THA and annually thereafter, until the last follow-up. At clinical evaluations, patients were assessed using the Harris Hip Score, the Merle d’ Aubigne and Postel Hip Score, the 12-item short-form health survey questionnaire (SF-12) scale, and the Hip Dysfunction and Osteoarthritis Outcome Score. Pre- and postoperative limp severity (severity of limping was assessed using a 4-point ordinal scale) and limb length discrepancy (LLD) were recorded. All complications were reviewed.

### Bacteriological evaluation

Before THA, several tests were performed to rule out persistent hip infections. At 8 weeks before the surgery, all the patients had attempted suction. If there is no fluid in the joint, salt water was used for flushing. Complete blood cell count and erythrocyte sedimentation rate (ESR) and C-reactive protein (CRP) levels were measured before surgery. Perioperative aspirates, smears, and excised specimens were collected for aerobic, anaerobic, and TB bacilli growth culture. Intraoperative frozen sectioning of suspicious tissue was performed. Pathologic examination is used to determine evidence of bacterial or tuberculous infection. If there were radiolucent areas on preoperative radiography, intraoperative biopsy was performed and cultured. Antibiotics were not given perioperatively until deep tissue material was obtained for culture. Cefuroxime was administered intravenously for 2 days at a daily dose of 4.5 g.

### Surgical technique

One-stage THA without cement was performed in all patients; 6 hips underwent cementless THA with transverse subtrochanteric shortening osteotomy. Four professional surgeons performed all the operations. All procedures were performed via the posterolateral approach, with the patient in a lateral position. Surgical procedures of the posterior lateral approach are well described in the literature [13]. The acetabular component (Pinnacle; DePuy Synthes, Warsaw, IN, USA) was fixed only by press-fit in 90 hips; in the remaining 11 hips, one or two screws were added to augment fixation.

If the acetabular bone was deficient, the resected femoral head was utilized to provide adequate coverage of the acetabular cup. The median diameter of the acetabular cup was 48 mm (range, 40–60). The femoral head size was 22 mm in 6 hips, 28 mm in 2 hips, and 32 mm in 93 hips. Ceramic-on-ceramic material was used in 74 hips, while metal-on-poly material was used in 27 hips. Cementless modular femoral stem (S-ROM, DePuy Synthes) was used in 34 hips, while others (Taper, DePuy Synthes) were used in 20 hips. In the hips that had cementless fixation, the femoral components were inserted with a press-fit. Although most of the anatomical structures of the hips have changed, the bone mass was good, probably because the patients were young.

All patients were encouraged to start exercising their limbs as early as possible after surgery.

### Radiographic analysis

Anteroposterior and lateral radiographs of the hip, full-length view of the lower extremities, and three-dimensional computed tomography of the hip were taken at each follow-up visit. Mu et al. reported the measuring method of cup inclination [14]. One hundred one patients had adequate preoperative and postoperative imaging for review. For six patients who had undergone transverse subtrochanteric shortening osteotomy, limb lengthening was measured on radiographs by subtracting the amount of femoral shortening from the amount of translation of the greater trochanter tip postoperatively [15]. The criteria described by Masonis et al. [16] assessed the bone healing at the osteotomy site using postoperative imaging. Serial radiographs were evaluated for evidence of component migration, heterotopic ossification, osteolysis, and so on. Seven zones were defined around the femoral component as described [17].

The acetabular component was diagnosed as loosening when the position of the acetabular component was changed or when a continuous radiolucent line was > 2 mm wide in both anteroposterior and lateral radiographs [18, 19]. Engh et al. reported the use of imaging analysis to assess femoral component looseness [20]. The extent of femoral stem osteointegration was classified as “bone ingrown,” “fibrous stable,” or “loose” as described [20]. The definition of femoral component submergence is reported in the literature [21].

### Statistical analysis

Results are reported as mean values with ranges. Differences in pre- and postoperative continuous variables were assessed using two-sided, paired Student’s *t* test. *P* < 0.05 was considered significant. Kaplan-Meier survival analysis was used to assess the time (1) until revision for any reason for any component or (2) until revision for radiographic loosening for any component. SPSS 19.0 was used for statistical analysis. (IBM, Armonk, USA).

## Results

Of the initial 108 hips in 105 patients, 2 patients (4 hips) had failed postoperative follow-up due to loss of contact information. Two patients (3 hips) died at 4 years after surgery as a result of causes unrelated to THA. The remaining 101 hips in 101 patients (51 men), all of whom underwent unilateral THA, were analyzed in this study. The patients had a mean age of 52.3 years (range, 24–79) at the time of surgery. The mean body mass index was 23.3 kg/m^2^ (range, 18.2–33.3). The mean interval between the resolution of active hip infection and arthroplasty was 24 years (range, 11–43).

### Pre- and perioperative results

Preoperative complete blood cell counts, ESR, and CRP levels in all patients were in the normal range. Mean operative time was 105 min (range, 45–175), and mean intraoperative blood loss was 400 ml (range, 200–700). Mean hospital stay was 8.5 days (range, 6–16). All intraoperative tests were negative for bacterial or tuberculous infection. Histology of frozen sections collected during surgery was negative for bacterial and tuberculous infection.

### Postoperative results

The mean duration of follow-up was 6.1 years (range, 2.1–9.6). During this period, none of the patients experienced infection recurrence in the hips treated by unilateral THA. Mean Harris Hip Score improved significantly from 48.5 points (range, 21–71) before surgery to 90 points (range 70–98) after surgery. Mean modified MAP Hip Score improved significantly from 5.5 points (range, 2–10) before surgery to 15.4 points (range, 12–19) after surgery. Similarly, the Hip Dysfunction and Osteoarthritis Outcome Score as well as the SF-12 score improved significantly between before and after surgery.

Preoperative LLD was 3.4 cm (range, 1.5–7), while postoperative LLD was 1.1 cm (range, 0.5–2.2) (Table 1). Postoperative LLD in all hips was < 1 cm in 43 cases, between 1 and 2 cm in 49 cases and between 2 and 3 cm in 9 cases. By the end of follow-up, none of the hips showed LLD > 3 cm. At 2-week follow-up, one patient complained that ipsilateral limb length was shorter on the operated side than on the other side, but no difference was found upon physical measurement. The same patient did not report shorter LLD at 6-month follow-up.

**Table 1:** Clinical outcomes

Indicator	Preoperative	Postoperative	*P*
Harris Hip Score			
Mean (range), points	48.5 (21–71)	90 (70–98)	< 0.01
Rating (no. of hips)			
Excellent (90–100 points)	0	53	
Good (80–89 points)	0	44	
Fair (70–79 points)	5	4	
Poor (< 70 points)	96	0	
Modified MAP			
Mean (range), points	5.5 (2–10)	15.4 (12–19)	< 0.01
Limp (no. of hips)			
Severe	24	0	
Moderate	48	4	
Slight	29	30	
None	0	67	
Limb length discrepancy			
Mean (range), cm	3.4 (1.5–7)	1.1 (0.5–2.2)	*P* < 0.01
Distribution (no. of hips)			
< 1	0	43	
1–2	11	49	
2–3	26	9	
3–4	15	0	
4–5	41	0	
> 5	8	0	
SF-12			
PCS	9.1 (5–17)	21.0 (18–25)	< 0.01
MCS	12.7 (8–20)	24.5 (21–29)	< 0.01
Hip Dysfunction and Osteoarthritis Outcome Score			
Symptoms	8.9 (4–13)	16.3 (14–20)	< 0.01
Pain	14.1 (5–24)	36.1 (34–39)	< 0.01
Daily living	28.1 (19–36)	58.9 (55–66)	< 0.01
Sports and recreational activities	5.1 (3–7)	12.9 (10–15)	< 0.01
Quality of life	4.5 (1–8)	12.3 (12–1)	< 0.01

Values are expressed as *n* or mean (range)

*MAP* Merle d’Aubigne and Postel, *MCS* mental component summary, *PCS* physical component summary, *SF-12* 12-item short-form health survey questionnaire

Mean leg prolonging was 2.1 cm (range, 1.1–3.9) postoperatively. Among the 34 patients who experienced postoperative limp, 4 had moderate limp, 30 had slight limp, and 0 had severe limp. Severe nonoperative limb pain caused by osteoarthritis of the hip may be one of the causes of the 4 patients (4 hips) of moderate limp.

### Radiographic results

**Fig. 1:**
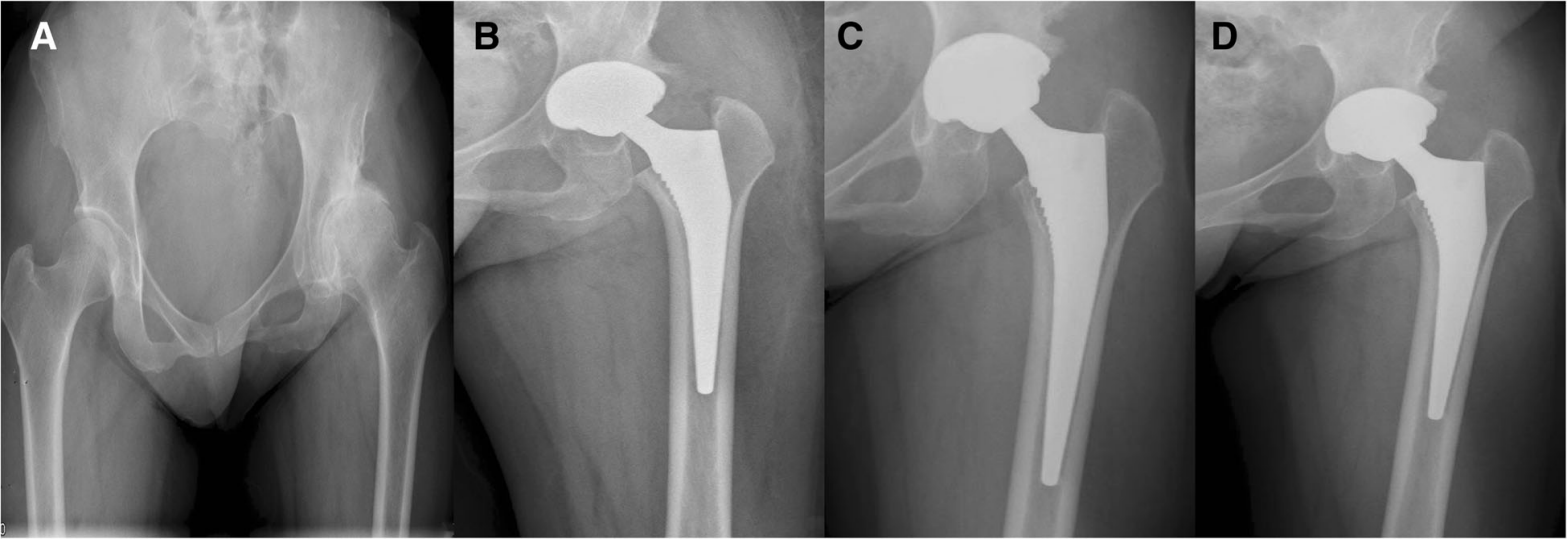
Radiographs of a 53-year-old woman with unilateral osteoarthritis secondary to hip pyogenic infection. **a** Preoperative anteroposterior view. **b** Postoperative imaging. Total hip arthroplasty is used to reconstruct the hip at the anatomical central level. **c** At 4-year follow-up, no radiolucent lines around the femoral stem were found. **d** At 7-year follow-up, no subsidence or loosening was found, and the femoral and acetabular components were considered to be stable

Solid bone union at the osteotomy site was achieved in 6 hips without complications; the mean union time was 6 months (range, 3–9). One hip had progressive radiolucent lines around the proximal femoral stem (zones 1 and 7), which leads to the progressive sinking of the femoral shaft and loosening of the femoral component. Revision surgery was performed at 2.2 years after initial THA, with a fully porous-coated stem, and the new femoral component was stable. One patient had loosening of the acetabular cup at postoperative 5.5 years, and the acetabular component was revised. By the end of follow-up, all acetabular components were found to be in situ and radiographically stable (Fig. 1). Follow-up radiographs of 19 hips revealed little focal osteolysis in the greater trochanter, but no surgery was required. During follow-up, asymptomatic heterotopic ossification in 5 hips was observed on radiographs: 3 hips were assigned to class I of the Brooker classification system [22] and 2 hips were assigned to class II.

### Complications

There were seven cases of femoral fracture, including five cases of distal femur and two cases of proximal femur. These fractures did not involve displaced cracks during bone preparation. After treatment with cerclage cables, fractures healed without further sequelae. Four patients (4 hips) experienced postoperative dislocation once. One patient received closed reduction, while the others were observed within 9 months after exponential surgery and were successfully treated with open reduction and internal fixation. The dislocation did not recur.

Three patients (3 hips) experienced transient sciatic nerve palsy with weak ankle dorsiflexion and foot numbness. The three patients with nerve palsy were treated with drugs and returned to normal after 8 months without any sensory or motor deficit. No evidence of deep venous thrombosis, infection, or polyethylene wear was found during follow-up.

### Survival analysis

**Fig. 2:**
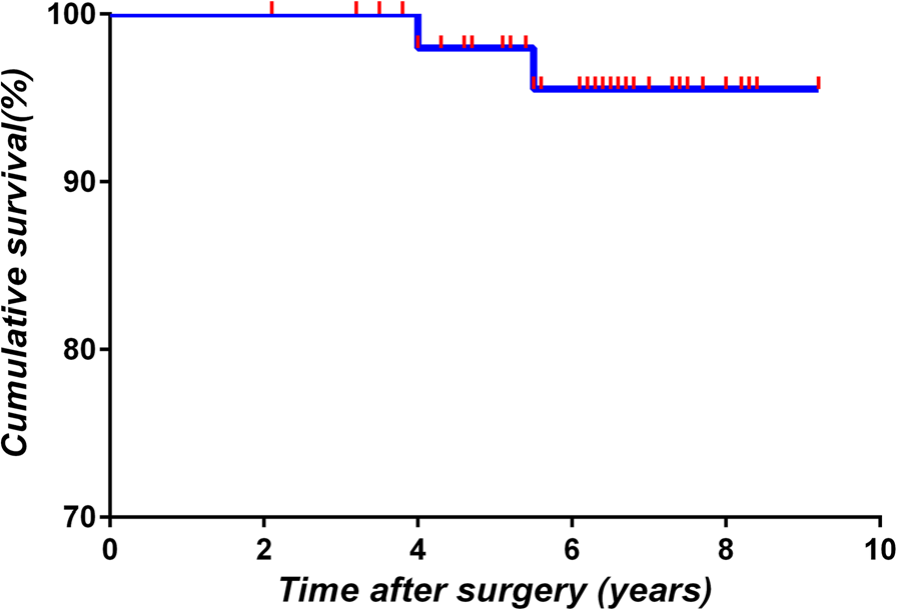
Kaplan-Meier analysis of time without radiographic loosening for any component

At 5.5 years postoperation, the Kaplan-Meier rate of survival without revision for any reason was 97.5% (95% confidence interval 90–99). The same results were obtained for the Kaplan-Meier rate of survival without revision for radiographic loosening for any component (Fig. 2).

## Discussion

THA in patients with hip pyogenic infection is challenging because of the risk of postoperative reinfection. Reducing the femoral head into the true acetabulum and maintaining it in position are difficult because of severe soft tissue contracture, persistent joint stiffness, coxa vara, anatomic abnormalities of the bone and soft tissue, abnormality in the neurovascular structures, and LLD. This has led clinicians to question whether and when THA should be attempted in a hip with a history of bacterial infection. One study [6] recommended performing THA only after infection remains quiescent for more than 10 years. Consistent with this and other studies [4, 6], we did not observe a single case of infection recurrence in our cohort of patients undergoing THA after more than 10 years of infection quiescence.

Our findings strengthen the existing evidence that THA can be effective for treating quiescent septic hip arthritis, with minimal adverse consequences, for instance, implant failures and infections (Table 2). Our results are consistent with the reinfection rate of 1.2% (2 of 170) in a large study of THA [4]. Some studies have reported higher reinfection rates [4, 11, 23], but these studies involved patients who underwent surgery after longer quiescence periods.

**Table 2:** Summary of published studies examining total hip arthroplasty in patients with quiescent septic arthritis infection of the hip

Study	No. of hips (patients)	Mean interval between presentation and operation, years	Mean follow-up, months	Rate of postoperative infection, *n* (%)	Complications (*n*)
Our study	101 (101)	24	75	0 (0)	Heterotopic ossification (5), dislocation (4), osteolysis (19), femoral fracture (7)
Papanna et al. [26]	7 (7)	4	66	0 (0)	Heterotopic ossification (2), dislocation (1)
Kim et al. [4]	170 (161)	33	124	2 (1.2)	Heterotopic ossification (16), dislocation (0), osteolysis (97), femoral fracture (3)
Laforgia et al. [23]	42 (38)	33	60	4 (9.5)	
Jupiter et al. [11]	24 (24)	27	43	1 (4.2)	
Bauer et al. [30]	9 (9)	5	60	0 (0)	

**Fig. 3:**
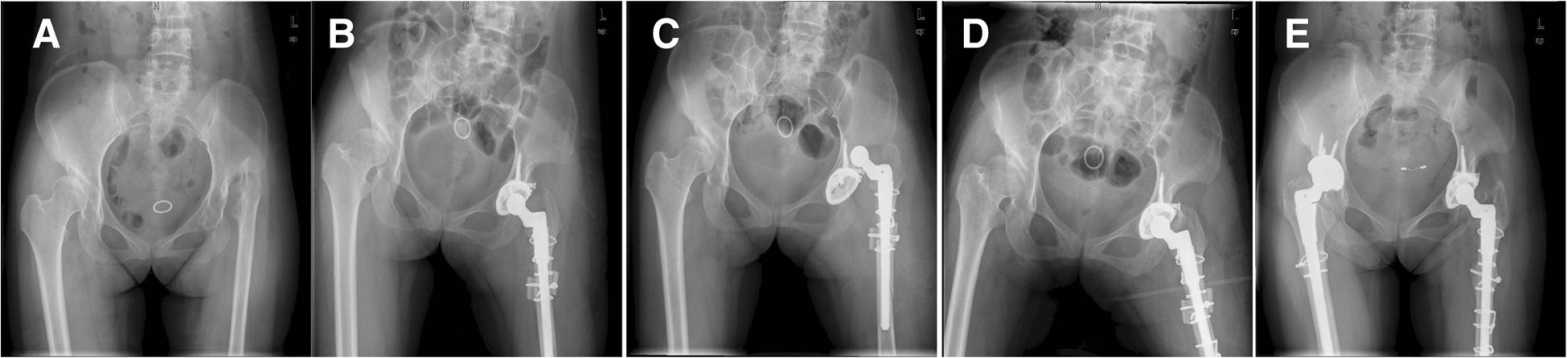
Radiographs of a 43-year-old woman with unilateral osteoarthritis secondary to hip pyogenic infection. **a** Preoperative anteroposterior view. **b** Postoperative radiographic image at 1 day. Total hip arthroplasty was performed combined with transverse osteotomy in the left hip. Fixation was conducted with wire and screw. **c**, **d** Postoperative anteroposterior view showed dislocation and distal fracture of the prosthesis at 3 days, which was treated successfully through open reduction and internal fixation. No dislocation recurred. The cause was an accidental fall. **e** At 9-year follow-up, the Harris Hip Score was 95. No loosening of components was found, and the femoral stem was judged to be stable with bone ingrowth. Total hip arthroplasty was performed combined with transverse osteotomy in the right hip. Fixation was conducted with wire and screw

Placing acetabular components in an anatomical position promotes long-term stability of acetabular and femoral components. In our study, all acetabular components were implanted in the acetabulum. After 5.5 years, only 1 hip (0.99%) developed aseptic loosening of the acetabular component. This loosening rate is substantially lower than the rate of 15.3% (26 of 170 hips) in another study [4]. However, the dislocation rate of our study (3.9%) was higher than the rate in that previous work (0%) [4]. No stem loosening or non-union was observed in these patients (Fig. 3).

Anatomical placement of acetabular components may result in nerve damage in severe osteoarthritis that occurs secondary to hip pyogenic infection. Indeed, 3 hips (the 3 hips showed leg lengthening of 3.8, 3.9, and 3.7 cm, respectively) in our study suffered transient nerve injuries, probably because of the substantial leg lengthening, lack of surgeon experience, and relatively long surgery. By the last follow-up, all nerve injuries had healed without further complications. We suggest that leg lengthening > 3.5 cm increases the risk of nerve injury, which would be consistent with previous work [24].

In our study, osteolysis accounted for 18.8% (19 hips). This rate was lower than that reported in a previous study (using cemented THA) of 58.5% (72 out of 123 hips) [4]. The results are the same as in previous studies [25]. In addition, the proportion of heterotopic ossification was 4.95% (5 hips), which was also lower than that reported in previous studies [4, 26], and the proportion of heterotopic ossification in these studies was 28.5% (2 out of 7 hips) and 9.4% (16 out of 170 hips), respectively.

Compared with the cemented THA, cementless THA had the following advantages: periprosthetic joint infection (PJI) rate was low [27]; the prosthesis had a higher survival rate [28]; although the bone is loosening, there is no bone loss, which is in contrast to the obvious osteolysis in bone cement implants [25]; and there is no risk of adverse events related to cement, such as cardiopulmonary complications [29].

Our results should be interpreted with caution given that this was a retrospective evaluation without a control group. In addition, we were unable to compare outcomes between uni- and bilateral THA.

## Conclusion

Cementless THA can effectively treat patients with a quiescent period of infection of the hip of more than 10 years, resulting in good functional outcomes and fewer complications. Risk of infection recurrence after THA in these patients seems extremely low.

## Abbreviations

CRP: C-reactive protein
ESR: Erythrocyte sedimentation rate
LLD: Limb length discrepancy
MAP: Merle d’Aubigne and Postel
MCS: Mental component summary
PCS: Physical component summary
PJI: Periprosthetic joint infection
SF-12: 12-item short-form health survey questionnaire
TB: Tuberculosis
THA: Total hip arthroplasty
